# Persistence and Transmission Dynamics of *Babesia ovis* After Imidocarb Dipropionate Treatment: Evaluation via Blood Transfusion and Tick Infestation

**DOI:** 10.3390/pathogens15010007

**Published:** 2025-12-20

**Authors:** Recep Firat, Mehmet Can Ulucesme, Arda Eyvaz, Mehmet Alatas, Munir Aktas, Onur Ceylan, Ferda Sevinc, Sezayi Ozubek

**Affiliations:** 1 Department of Parasitology, Faculty of Veterinary Medicine, University of Firat, 23119 Elazig, Türkiye; rkupik@gmail.com (R.F.); mculucesme@firat.edu.tr (M.C.U.); ardaeyvaz1997@gmail.com (A.E.); vetmehmetalatas@gmail.com (M.A.); maktas@firat.edu.tr (M.A.); 2 Department of Parasitology, Faculty of Veterinary Medicine, University of Selcuk, 42130 Konya, Türkiye; onurceylan@selcuk.edu.tr (O.C.); fsevinc@selcuk.edu.tr (F.S.)

**Keywords:** *Babesia ovis*, blood transfusion, imidocarb dipropionate, parasite elimination *Rhipicephalus bursa*

## Abstract

*Babesia ovis* is a significant tick-borne parasite of sheep, capable of causing both acute disease and long-lasting, low-grade infections. Imidocarb dipropionate (IMDP) is commonly used against babesiosis, yet whether it can completely eliminate *B. ovis* remains uncertain. In this study, we examined whether the parasite persists after treatment and whether such residual infections can still be transmitted. Three sheep were experimentally infected, treated with IMDP once clinical signs appeared, and then monitored for 180 days by microscopy, nested PCR, and iELISA. Fever and microscopic parasitemia resolved soon after treatment, but nPCR intermittently detected parasite DNA for several weeks. By day 180, all treated sheep were negative by nPCR and microscopy, while two still showed detectable antibodies. Blood collected at this time was transfused into naïve sheep. Two of the three recipients showed nPCR positivity at scattered time points and later seroconverted while showing no clinical signs. In contrast, *Rhipicephalus bursa* ticks that fed on the treated donors neither acquired the parasite nor transmitted it to recipients, likely because post-treatment parasitemia remained below the acquisition threshold. Overall, these results indicate that IMDP controls clinical disease but may not fully clear *B. ovis*, allowing silent transmission through blood despite negative routine tests.

## 1. Introduction

*Babesia ovis* is a tick-borne parasite found across many regions, including southern Europe, Africa, the Middle East, and parts of Asia [1,2]. It mainly infects sheep and causes ovine babesiosis, a disease that can range from mild lethargy to severe anemia, high fever, and weight loss [3,4,5]. The parasite multiplies in sheep red blood cells and completes its sexual stage in the tick *Rhipicephalus bursa*, which plays a central role in maintaining the infection in endemic areas [6,7]. Goats can also be infected, but they rarely show clinical signs, suggesting a degree of species-related resistance [8]. In sheep, however, *B. ovis* remains an important cause of both acute and chronic disease and can lead to substantial economic losses in areas where the vector is common [1,9]. The antipiroplasmic drugs can effectively manage clinical signs; however, the complete clearance of the parasite is still a main obstacle in the control of ovine babesiosis [10]. At the moment, the only practical way to control the disease is by reducing tick numbers with acaricides, since there is still no vaccine or any other long-term preventive option [5].

Imidocarb dipropionate (IMDP) is the drug most often used to treat babesiosis and typically reduces parasitemia and improves clinical status [11]. It is an aromatic diamidine, with use mostly for large *Babesia* infections [12]. Although IMDP is effective, it has considerable safety issues. It has hepatotoxic and nephrotoxic effects at high doses and can induce serious side effects at therapeutic doses, including colic, diarrhea, and the excessive production of salivary secretions [13,14,15]. Moreover, IMDP residues were also found in sheep and goat milk [16], as well as in different bovine tissues [17], which could be a food safety concern. An additional important question is whether IMDP can completely clear blood parasites from the host. In other species of *Theileria* and *Babesia*, such as *Theileria equi*, *T. haneyi* [18], and *Babesia negevi* [19], it has recently been documented that treated animals may still harbour latent infections and that this may persist despite the absence of detectable parasitemia. Therefore, achieving complete parasite elimination is desirable not only to prevent clinical relapse and transmission, but also to limit repeated drug exposure and reduce potential residue-related food safety concerns. This raises concerns regarding whether *B. ovis* can persist after treatment and remain transmissible through blood transfusion or tick vectors, potentially leading to disease recurrence and continued spread. Previous studies have examined the use of IMDP in *B. ovis* infections [20]; however, these were conducted using conventional diagnostic methods without molecular or serological confirmation. Given the increasing evidence of latent infections in other *Babesia* species post-treatment, it is essential to reassess IMDP efficacy with current diagnostic approaches.

In this context, serological tests based on *B. ovis* secreted antigen 1 (BoSA1), a well-characterized and immunodominant protein, are useful for identifying persistent or low-level infections [3,21]. This study aims to assess the long-term elimination of *B. ovis* following IMDP treatment by evaluating its potential transmission through blood transfusion and tick infestation. By addressing a critical gap in our understanding of post-treatment persistence and transmission potential, this study provides valuable insights into the efficacy of IMDP treatment, the epidemiology of *B. ovis*, and broader implications for disease control in endemic regions.

## 2. Materials and Methods

### 2.1. Experimental Animals

This study included a total of ten (*n* = 10) Akkaraman male sheep aged 6–8 months and free from tick-borne pathogens. Experimental animals were selected according to Ozubek et al. (2025) [4]. All sheep were subjected to a screening for *Theileria*, *Babesia*, *Anaplasma* and *Ehrlichia* genera by nested PCR (nPCR) before purchasing and only nPCR negative were screened thereafter (Supplementary Table S1). Serological examination of nPCR-negative sheep was then performed by an indirect ELISA (iELISA) based on recombinant *Babesia ovis* Secreted Antigen 1 (BoSA1) [21]. The study included animals that were negative by both molecular and serological methods.

Three sheep (#842, #953, #1006) were assigned to the infection–treatment group. The remaining seven sheep served as naïve recipients for either blood transfusion (#1763, #1751, #1759) or tick infestation (#4577, #4513, #4520). One additional splenectomized sheep (#Donor) was used solely as a source of fresh *B. ovis*–infected blood for experimental inoculation. The small number of experimentally infected animals follows previously published controlled infection studies and reflects the ethical requirement to minimize animal use in pilot transmission experiments.

All procedures were approved by the Firat University Animal Experiments Ethics Committee (protocol 2023/12-05). All animals were housed in vector-free indoor facilities, kept under routine husbandry conditions, and monitored daily for general health. Sheep were provided ad libitum access to fresh water and standard sheep feed.

### 2.2. Infection of Sheep

**Figure 1:**
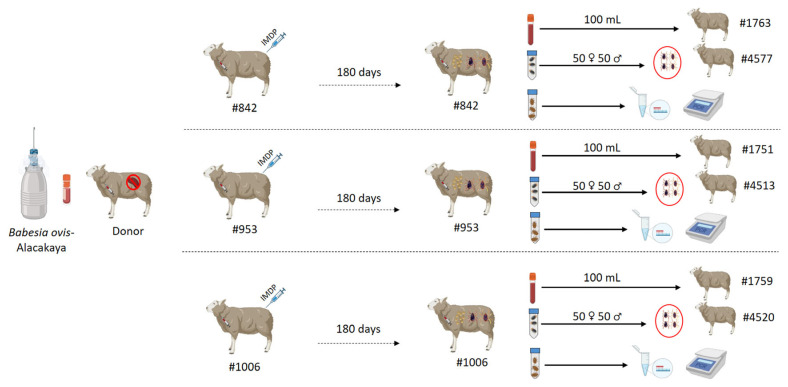
Schematic overview of the experimental design, illustrating the use of sheep for infection studies involving both blood transfusion and tick infestation.

In one sheep (#Donor) selected for experimental infections, the splenectomy was performed according to the method described by [10] to obtain fresh blood infected with *B. ovis*. After post-operative care, the splenectomized sheep (#Donor) was intravenously inoculated with 15 mL of cryopreserved *B. ovis*-Alacakaya stabilate [3,4,8]. The donor sheep was daily monitored for clinical signs and parasitemia levels by microscopic examination. Peripheral blood smears were obtained by ear-tip from experimentally infected sheep for microscopic examination. Blood smears were stained with Giemsa, and screened with a 100X oil immersion objective for infected erythrocytes. The PPE (percentage of parasitized erythrocytes) was determined from at least 20 microscopic fields of the edge regions of the blood smear sample using the method defined by [22]. When parasitemia reached an estimated 5%, 90 mL of infected blood was collected into vacutainer K_2_EDTA tubes (10.0 mL each). This blood was then intravenously administrated to three experimental sheep (#842, #953, and #1006) for the purpose of establishing experimental infection (30 mL per animal) (Figure 1).

### 2.3. Treatment of Sheep and Sample Collection

Following experimental infection, the three infected sheep (#842, #953, and #1006) were monitored daily for clinical signs and parasitemia (PPE) using microscopic examination. Once clinical symptoms developed, a single dose of 1.2 mg/kg IMDP was administered [10] intramuscularly between days 10 and 14 post-infection. After experimental infection, body temperature was recorded daily for the first 30 days and then every three days until day 180. Throughout the study, clinical status was routinely assessed, and blood samples were collected into EDTA and serum tubes for molecular and serological analysis.

### 2.4. Long-Term Detection of B. ovis DNA and Antibody Response in Experimentally Infected Sheep

Following experimental infection, microscopic, serological, and molecular methods were employed to monitor *B. ovis*. Two-step process of molecular detection was based on nPCR, first amplifying with the Nbab1F/Nbab1R primer set [23] and then with the BboF/BboR primer set [24]. These analyses were carried out to assess the persistence of *B. ovis* DNA in experimentally infected sheep over time. A recombinant BoSA1-based iELISA was used for serological monitoring to assess the antibody response [21]. The rBoSA1 protein used as the antigen in the iELISA was expressed and purified in *Escherichia coli*. rBoSA1 at 2 μg/mL in carbonate-bicarbonate buffer (pH 9.6) was used to coat ELISA microplates overnight at 4 °C; microplates were then blocked followed by the incubation with diluted (1:100) sheep sera with horseradish peroxidase-conjugated anti-sheep IgG (Sigma-Aldrich, St. Louis, MO, USA). A chromogenic substrate [2,2′-azinobis (3-ethylbenzothiazoline-6-sulfonic acid)] (Sigma, Louis, MO, USA) was added for the reaction, and optical densities (OD) were read at 450 nm. As determined by differentiation of OD of positive and negative samples, the cut-off value was defined as the average OD of negative control sera plus three standard deviations.

### 2.5. Evaluation of B. ovis Elimination via Blood Sub-Inoculation and R. bursa Infestation

To assess the elimination of *B. ovis* at the end of the 180-day monitoring period, 100 mL of blood collected from experimentally infected sheep (#842, #953, and #1006) was intravenously administered to three naive sheep (#1751, #1759, and #1763). These recipient sheep were then monitored for 100 days using microscopic, serological, and molecular methods to detect any potential infection.

In addition to blood sub-inoculation, tick transmission was also evaluated. To allow parasite acquisition by ticks, experimentally infected sheep (#842, #953, and #1006) were initially infested with *Babesia* sp.–free *R. bursa* larvae (0.1 g) and adult ticks (50 females and 50 males) derived from a laboratory colony of *R. bursa* [3,25]. Following infestation, engorged nymphs and engorged female ticks were collected and incubated under controlled conditions (27 °C and 85% relative humidity). This incubation allowed molting of engorged nymphs into unfed adult ticks and oviposition by engorged females. Unfed adult ticks that emerged from engorged nymphs were subsequently used for transmission experiments. For this purpose, 50 female and 50 male unfed adult ticks derived from each experimentally infected sheep (#842, #953, and #1006) were placed onto three naïve sheep (#4513, #4520, and #4577) to assess post-treatment tick-borne transmission of *B. ovis* (Figure 1; Table 1). These newly infested sheep were monitored for 100 days, during which clinical, microscopic, serological, and molecular assessments were performed. Additionally, larvae emerging from engorged females and their carcasses were analyzed using nPCR to assess potential transovarial transmission of *B. ovis* [4]. Briefly, after the completion of the oviposition period, half of the engorged female ticks were dissected using a scalpel and transferred into Eppendorf tubes, where they were pulverized in liquid nitrogen. Similarly, 0.1 g of larvae collected from these females was also ground in liquid nitrogen. Both samples were stored at −20 °C until genomic DNA extraction. Genomic DNA was extracted using the PureLink™ Genomic DNA Mini Kit (Invitrogen, Carlsbad, CA, USA) according to the manufacturer’s instructions. This kit was used for DNA extraction from both blood and tick samples in this study.

### 2.6. Statistical Analysis

This study was designed as an exploratory experimental infection model and did not aim to perform inferential statistical comparisons. Therefore, no hypothesis-driven statistical tests were applied. Descriptive statistics (range and frequency of nPCR positivity, PPE values, and iELISA optical densities) were used to summarize the data.

## 3. Results

### 3.1. Clinical Progression and Treatment of Experimentally Infected Sheep

Three sheep were experimentally infected and treated, three naïve sheep received blood transfusions, and three sheep were used for tick infestation experiments.

The clinical course of *B. ovis* infection was monitored daily following experimental infection with the *B. ovis* Alacakaya stabilate. In sheep #842, fever was first detected on days post-infection (DPI) 5, reaching 40.9 °C, with the initial presence of parasites confirmed via microscopic examination of blood smears. Parasitemia lasted for five days, reaching a maximum PPE of 0.5%. The highest recorded fever was 41.9 °C, which lasted six days. Clinical signs, including fever, anemia, and jaundice, were observed, and the sheep were treated with 1.2 mg/kg IMDP on DPI 11.

In sheep #953, fever developed earlier, rising to 41.3 °C on DPI 2, with the first detection of *B. ovis* parasites microscopically on DPI 3. The fever peaked at 42.0 °C and persisted for ten days, while PPE lasted six days, with a maximum PPE of 0.8%. The animal exhibited fever, anemia, and jaundice, necessitating treatment with IMDP on DPI 10.

In sheep #1006, the clinical course was notably milder than in the other two sheep (#842, and #953). The febrile phase lasted only one day, and PPE was detectable for just two days. Fever peaked at 40.5 °C on DPI 11, with parasites observed microscopically. The following day, PPE reached 0.1%, and the animal was treated with IMDP on DPI 14. Unlike the other sheep, only mild anemia was observed, and no additional clinical symptoms were noted.

In addition, no adverse effects were observed following IMDP treatment in any of the experimentally infected sheep. All treated animals tolerated the drug well, with no signs of toxicity recorded throughout the study.

### 3.2. Post-Treatment Blood Transfusion Can Transmit B. ovis, but R. bursa Does Not Acquire or Transmit the Parasite

Presence of *B. ovis* DNA in experimentally infected sheep was monitored by nPCR during the course of the study. Between DPI 1 and 6, *B. ovis* DNA was detected in all infected sheep. Following treatment, nPCR detection of *B. ovis* DNA was intermittent, and parasite DNA was last detected on DPI 144, 70, and 135 in sheep #842, #953, and #1006, respectively. Detection patterns were not uniform among the three sheep. On nPCR, post-treatment *B. ovis* DNA persisted intermittently, indicating that the parasite was not completely eliminated. All three sheep developed anti-*B. ovis* antibodies serologically between DPI 8 and 10. Antibody responses were sustained up to 180 DPI in sheep #953 and #1006, however in sheep #1006 antibody titers decreased and were undetectable at DPI 153. On day 180, *B. ovis* DNA could not be detected in any of the three infected sheep (#842, #953 and #1006), but serological responses remained observable in two sheep (#842, and #953).

**Figure 2:**
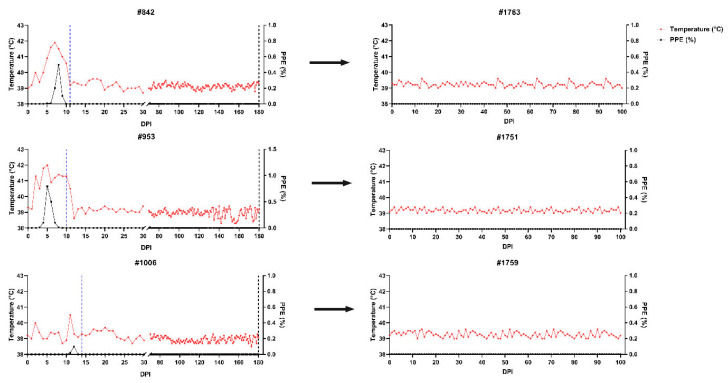
Body temperature and parasitemia dynamics in experimentally infected (**left**) and transfused (**right**) sheep. Donor sheep (#842, #953, #1006) developed fever and detectable parasitemia, while transfused recipients (#1763, #1751, #1759) showed no significant parasitemia or fever. The blue dashed lines indicate IMDP treatment, and black dashed lines represent the time of blood transfusion.

**Figure 3:**
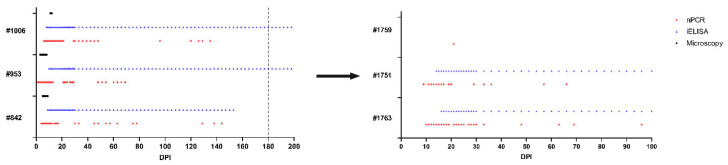
Detection of *B. ovis* infection dynamics using microscopy, iELISA, and nPCR. On day 180, experimentally infected sheep (**left**) were infested with *Babesia*-free *R. bursa* larvae and adult ticks, and their blood was transfused into recipient sheep (**right**).

**Figure 4:**
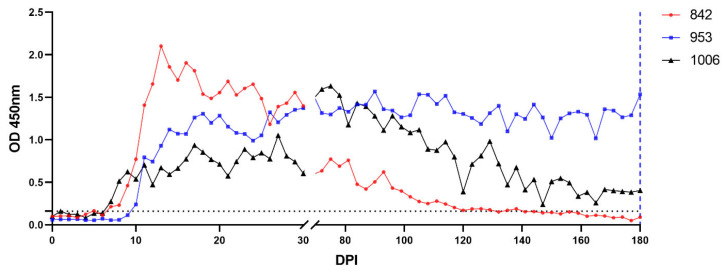
Serological response to *B. ovis* infection in experimentally infected sheep measured by BoSA1-based iELISA. The blue dashed lines at day 180 indicates the time of tick infestation and blood collection. The dotted horizontal black line represents the cut-off value for seropositivity.

**Figure 5:**
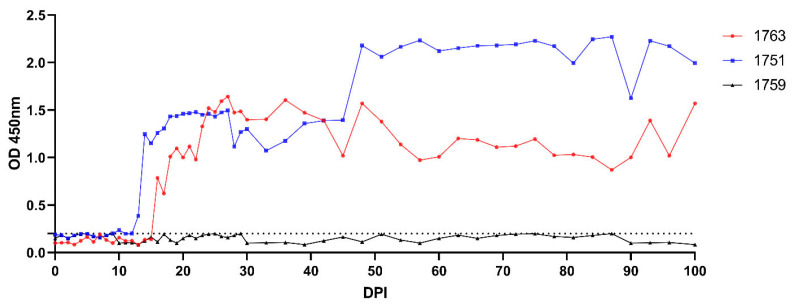
Serological response in transfused sheep measured by BoSA1-based iELISA. The dotted horizontal black line represents the cut-off value for seropositivity.

To determine whether *B. ovis* was eliminated after IMDP treatment, 100 mL blood from sheep #842, #953, and #1006, respectively, was transfused into naïve sheep #1763, #1751, and #1759. The sheep that received them were evaluated at 100 days using clinical, microscopic, molecular, and serological techniques. None of the transfused sheep developed the typical clinical signs of babesiosis (fever, jaundice, hemoglobinuria) and *B. ovis* was not detected microscopically in any of the recipient animals. Indeed, nPCR analysis revealed that *B. ovis* DNA persisted in two of the three transfused sheep, indicating that transmission via blood transfusion was successful even in the absence of clinical disease. For sheep #1763, *B. ovis* DNA was first observed at DPI 10 and was intermittently detectable until DPI 96. Antibodies to *B. ovis* developed by DPI 16 and remained positive until the 100 DPI. Likewise, *B. ovis* DNA was detected only in sheep #1751 from DPI 9 onwards, at which point it exhibited a pattern closely mimicking that of sheep #1763, but it was detectable only until DPI 66. Antibodies were detected from DPI 14 and continued to be present at day 100. In contrast, *B. ovis* DNA was only found once in sheep #1759, at DPI 21, with no detectable serological response during the study period (Figure 2, Figure 3, Figure 4 and Figure 5).

The experimental infection of sheep revealed that *Babesia* sp.-free *R. bursa* larvae (0.1 g), and adults containing (50 females and 50 males) were tested to measure any potential tick transmission. Each day, we monitored tick development. Engorged male and female ticks were collected 8–14 days post-infestation. All engorged nymphs were gathered between 14 and 20 days post-infestation. Engorged ticks were kept in different environmental conditions to facilitate the emergence of adult *R. bursa* ticks from engorged nymphs and larvae from engorged females. To assess post-treatment transmission in more detail, naïve sheep #4577, #4513, and #4520 were infested with 50 male and 50 female unfed adult ticks derived from engorged nymphs. Sheep were followed for 100 days using microscopic, molecular (nPCR), and serological (iELISA) approaches. None of the recipient sheep (#4577, #4513, and #4520) showed any evidence of *B. ovis* transmission. Moreover, engorged male and female ticks collected from the same sheep (#4577, #4513, and #4520) were also analyzed by nPCR, and the test results were negative for *B. ovis*.

## 4. Discussion

This investigation assessed the persistence and possibility of *B. ovis* transmission after IMDP treatment (1.2 mg/kg) using blood transfusion and tick infestation as transmission pathways. The current study demonstrated that sheep were still able to harbor *B. ovis* DNA after treatment with IMDP, enabling *B. ovis* transmission through blood transfusion. However, tick infestation did not allow *R. bursa* to acquire or transmit *B. ovis*. These results suggest that IMDP administration may control clinical disease, but does not guarantee complete parasite clearance from blood that could otherwise lead to cryptic carriage responsible for ongoing transmission.

Imidocarb dipropionate has been widely used to manage babesiosis in diverse animal species, as it decreases parasitemia and clinical signs [11]. However, there are concerns about incomplete parasite clearance as some studies have shown that infections may persist despite treatment. In our study, all three sheep remained nPCR-positive after infection for various periods of time, with one sheep (#842) remaining positive for as long as 144 days after infection. Importantly, nPCR positivity was not continuous, indicating changes in levels of parasitemia or low-level replication of parasites. Correlation with previous research indicates the ineffectiveness that IMDP has in the complete clearance of *T. equi* infections in horses despite repetitive treatment regimens [18,26]. Similarly, *T. haneyi*-infected horses did not achieve chemosterilization with IMDP and treatment itself was noted to not always clear *T. equi* [18]. In addition, IMDP was unable to clear *B. negevi* in infected dogs, with parasite persisting for up to 7 months in dogs [19].

Serological analysis in our study provides evidence that *B. ovis* persists, with antibody responses remained detectable until day 180 in #842 and #953, while #1006 became seronegative after DPI 153. This suggests that the immune system can still recognize *B. ovis* antigens, even though no parasitemia can be detected. Long-term seropositivity has been documented in other protozoal infections with long-lived plasma cells contributing to antibody circuits despite an absence of antigenic stimulation. This further underscore the need of using both serological and molecular tests for post-treatment evaluation, as previously proposed with *T. equi* [27].

Although cELISA in combination with nPCR has been reported as a highly reliable method for confirming infection clearance following IMDP treatment in naturally infected horses [27], our study demonstrates that a negative nPCR and iELISA result does not necessarily indicate complete parasite elimination. This was evidenced by successful *B. ovis* transmission from an nPCR- and iELISA-negative donor sheep to a naïve recipient via blood transfusion. This finding suggests that some latent infections may persist below the detection threshold of current diagnostic tools, reinforcing the need for more sensitive molecular and serological approaches for post-treatment monitoring. The ability of subclinical carriers to silently transmit *B. ovis* through blood transfusion raises concerns about the potential for undetected carriers to perpetuate the parasite within endemic sheep populations. This emphasizes the need for improved diagnostic tools before blood transfusion practices, particularly in endemic areas.

A key finding of this study is that *B. ovis* was successfully transmitted via blood transfusion to naïve recipient sheep, even though donor animals (#842, #953, and #1006) showed no clinical signs. Nested PCR confirmed transfusion-mediated transmission in two out of three recipients (#1763 and #1751), where parasitemia persisted for up to 96 days. Notably, one recipient (#1759) tested nPCR-positive only once (DPI 21) and did not develop a detectable antibody response, suggesting a transient or non-productive infection. These findings align with previous studies on *T. equi*, where donor animals tested negative by microscopy but remained infectious at the molecular level, enabling silent transmission [28]. Similarly, in *T. haneyi*-infected horses, IMDP treatment failed to eliminate parasites completely, allowing transmission through blood transfusion [18]. A similar observation was reported in *B. negevi*-infected dogs, where IMDP treatment did not achieve complete parasite clearance, and infections persisted for up to seven months in some animals, despite the absence of detectable parasitemia [19]. These results highlight the potential risk of cryptic transmission in subclinical carriers and reinforce the need for a more comprehensive screening approach.

Although *B. ovis* transmission occurred via blood transfusion, tick infestation did not lead to parasite acquisition or transmission by *R. bursa*. Despite the experimental infestation of infected sheep with *Babesia* sp.-free *R. bursa* larvae and adults, none of the naïve sheep infested with derived ticks tested positive for *B. ovis* by nPCR or serology. Additionally, engorged ticks and eggs tested negative for *B. ovis* DNA, suggesting that *R. bursa* failed to acquire or transmit the parasite. A possible explanation is that parasitemia levels in our study may have been too low for effective acquisition by ticks, particularly after IMDP treatment. However, this outcome was expected, as previous studies have shown that even in untreated animals, *B. ovis* infections persisting for up to six months do not lead to successful parasite acquisition by *R. bursa* [3]. This suggests that even if *R. bursa* is a competent vector, transmission requires sufficiently high parasitemia levels in the host. IMDP treatment in our study likely suppressed parasitemia below the acquisition threshold, preventing transmission.

While this study provides valuable insights into *B. ovis* persistence and transmission after IMDP treatment, some limitations should be considered. Although the relatively small sample size may limit the generalizability of the findings and larger-scale studies are needed to validate these results, it is important to note that *B. ovis* was not completely eliminated in any of the three treated sheep. Parasitemia was monitored using nPCR, which, despite its high sensitivity, has a detection threshold that may have underestimated residual infections. More sensitive molecular techniques, such as quantitative PCR (qPCR) or digital PCR, could provide a better assessment of post-treatment parasite burden. Additionally, this study evaluated only a single-dose (1.2 mg/kg) IMDP protocol, based on previous recommendations for ovine babesiosis, but alternative dosing regimens, such as repeated or higher doses, were not explored. Given that some studies suggest that higher doses may enhance parasite clearance, further research should assess the effectiveness of different treatment protocols. Indeed, previous findings indicate that even high-dose IMDP treatment may not be sufficient to completely eliminate *B. caballi* and *T. equi* infections from healthy carriers [29]. The failure of IMDP to achieve sterile parasite clearance, as demonstrated in the present study, underscores the need to explore alternative therapeutic strategies that may more effectively eliminate *Babesia* infections. Moreover, recent in vitro studies have shown that alternative antiprotozoal compounds may offer greater efficacy against *Babesia* species than IMDP [30,31,32]. For instance, buparvaquone, a second-generation hydroxynaphthoquinone antiprotozoal drug commonly used to treat bovine theileriosis, has been demonstrated to be more effective than IMDP against *B. bovis* in vitro [30]. Furthermore, another study reported that the combination of buparvaquone and ELQ316 was superior to the ELQ316 and IMDP combination in inhibiting *B. bovis* and *B. bigemina* growth in vitro [31,32]. These findings suggest that further exploration of alternative treatment strategies, including buparvaquone-based therapies, may be warranted for *B. ovis* infections. Another limitation is that only one strain of *B. ovis*-Alacakaya was used, preventing an assessment of potential strain-dependent variations in IMDP efficacy. The *B. ovis*-Alacakaya strain used in this study was originally isolated from naturally infected sheep in eastern Türkiye, a region where ovine babesiosis is endemic, and has been used in previous experimental transmission and pathogenicity studies [3,4,8,33]. Since genetic differences in *Babesia* species can influence drug susceptibility, comparative studies using multiple *B. ovis* strains would help determine whether treatment outcomes vary by genotype.

In summary, this study demonstrates that while IMDP controlled clinical disease, it failed to achieve complete *B. ovis* clearance, as evidenced by intermittent nPCR positivity in donor sheep and successful transmission via blood transfusion. Serological responses persisted for up to 180 days, despite undetectable parasitemia, while *R. bursa* failed to acquire the parasite, likely due to low parasitemia levels. Our result suggest the need for improved diagnostics and alternative therapies to ensure complete parasite elimination.

## 5. Conclusions

Our study shows that the IMDP treatment does not eliminate *B. ovis*, as molecular evidence of persisting parasite DNA in IMDP-treated sheep was detected. Blood transfusion successfully transmitted the parasite to naïve sheep, confirming that subclinical carriers remain infection source. Although no evidence of transmission via *R. bursa* was found, this may be due to insufficient parasitemia levels post-treatment rather than the vector’s inability to acquire or transmit the parasite. Furthermore, this study demonstrates that clearance of parasites, as determined by standard diagnostic methods, may not be definitive, because blood from an nPCR and iELISA negative donor (#842) transmitted *B. ovis* to a naïve recipient (#1763). These findings emphasize the need for improved diagnostic tools, safer blood transfusion protocols, and further research into *B. ovis* transmission dynamics and drug efficacy. Future studies should focus on optimizing post-treatment monitoring strategies, reassessing IMDP dosing regimens, and exploring alternative therapeutic approaches to ensure complete parasite clearance.

## Figures and Tables

**Table 1:** Overview of IMDP-treated donor sheep and corresponding recipient animals in blood transfusion and tick-mediated transmission experiments.

	Transmission by Direct Blood Inoculation	
Donor Sheep	Inoculation Amount	Recipient Sheep
#842	100 mL-intravenous	#1763
#953	100 mL-intravenous	#1751
#1006	100 mL-intravenous	#1759
	**Transmission by Tick Feeding**	
**Donor Sheep**	***R. bursa* Adult Tick**	**Recipient Sheep**
#842	50 female 50 male	#4577
#953	50 female 50 male	#4513
#1006	50 female 50 male	#4520

“#” denotes the individual sheep identification number (animal ID/ear tag).

## Data Availability

The original contributions presented in the study are included in the article, further inquiries can be directed at the corresponding author.

## Supplementary Materials

The following supporting information can be downloaded at https://www.mdpi.com/article/10.3390/pathogens15010007/s1, Table S1: Primers and sequences used in this study for the detection of tick-borne pathogens [34,35,36].

## Abbreviations

The following abbreviations are used in this manuscript:

IMDP	Imidocarb dipropionate
nPCR	Nested PCR
iELISA	indirect ELISA
BoSA1	*Babesia ovis* Secreted Antigen 1
PPE	Percentage of parasitized erythrocytes
DPI	Days post-infection
mL	Milliliter
OD	Optical density

## References

[1] Ceylan O., Xuan X., Sevinc F. (2021). Primary tick-borne protozoan and rickettsial infections of animals in Turkey. Pathogens.

[2] Schnittger L., Ganzinelli S., Bhoora R., Omondi D., Nijhof A.M., Florin-Christensen M. (2022). The piroplasmida *Babesia*, *Cytauxzoon*, and *Theileria* in farm and companion animals: Species compilation, molecular phylogeny, and evolutionary insights. Parasitol. Res..

[3] Firat R., Ulucesme M.C., Aktaş M., Ceylan O., Sevinc F., Bastos R.G., Suarez C.E., Ozubek S. (2024). Role of *Rhipicephalus bursa* larvae in transstadial transmission and endemicity of *Babesia ovis* in chronically infected sheep. Front. Cell. Infect. Microbiol..

[4] Ozubek S., Ulucesme M.C., Ceylan O., Sevinc F., Aktaş M. (2025). The Impact of *Babesia ovis*-infected *Rhipicephalus bursa* larvae on the severity of babesiosis in sheep. Front. Cell. Infect. Microbiol..

[5] Stuen S. (2020). Haemoparasites—Challenging and wasting infections in small ruminants: A review. Animals.

[6] Yeruham I. (1995). Babesiosis in sheep: The ecology of the tick vector *Rhiphicepalus bursa*, transmission of *Babesia ovis*, immunological, clinical and clinico-pathological aspects. PhD Thesis.

[7] Yeruham I., Hadani A., Galker F. (1998). Some epizootiological and clinical aspects of ovine babesiosis caused by *Babesia ovis*—A Review. Vet. Parasitol..

[8] Ozubek S., Ulucesme M.C., Suarez C.E., Bastos R.G., Aktas M. (2024). Assessment of *Babesia ovis* pathogenicity in goats: Implications for transmission dynamics and host resistant. Front. Cell. Infect. Microbiol..

[9] Sevinc F., Xuenan X. (2015). Major tick-borne parasitic diseases of animals: A frame of references in Turkey. Eurasian J. Vet. Sci..

[10] Sevinc F., Turgut K., Sevinc M., Ekici O.D., Coskun A., Koc Y., Erol M., Ica A. (2007). Therapeutic and prophylactic efficacy of imidocarb dipropionate on experimental *Babesia ovis* infection of lambs. Vet. Parasitol..

[11] Mosqueda J., Olvera-Ramírez A., Aguilar-Tipacamú G., Cantó G. (2012). Current advances in detection and treatment of babesiosis. Curr. Med. Chem..

[12] Pudney M., Gray J.S. (1997). Therapeutic efficacy of atovaquone against the bovine intraerythrocytic parasite, *Babesia divergens*. J. Parasitol..

[13] Meyer C., Guthrie A.J., Stevens K.B. (2005). Clinical and clinicopathological changes in 6 healthy ponies following intramuscular administration of multiple doses of imidocarb dipropionate. J. S. Afr. Vet. Assoc..

[14] Baneth G. (2018). Antiprotozoal Treatment of canine babesiosis. Vet. Parasitol..

[15] Esmaeilnejad B., Samiei A., Hasani S.J., Anassori E., Tavassoli M., Mofidi S.K.M. (2025). A review of babesiosis caused by *Babesia ovis* in small ruminants. Trop. Anim. Health Prod..

[16] Belloli C., Lai O.R., Ormas P., Zizzadoro C., Sasso G., Crescenzo G. (2006). Pharmacokinetics and mammary elimination of imidocarb in sheep and goats. J. Dairy Sci..

[17] Tang Y., Yu N., Liu C., Han M., Wang H., Chen X., Kang J., Li X., Liu Y. (2022). Residue depletion of imidocarb in bovine tissues by UPLC-MS/MS. Animals.

[18] Sears K., Knowles D., Dinkel K., Mshelia P.W., Onzere C., Silva M., Fry L. (2020). Imidocarb dipropionate lacks efficacy against *Theileria haneyi* and fails to consistently clear *Theileria equi* in horses co-infected with *T. haneyi*. Pathogens.

[19] Salant H., Nachum-Biala Y., Zivotofsky D., Tzur T.E., Baneth G. (2024). *Babesia negevi* infection in dogs and response to treatment. Tick Tick-borne Dis..

[20] Hashemi-Fesharki R. (1977). Studies on imidocarb dihydrochloride in experimental *Babesia ovis* infection in splenectomized lambs. Br. Vet. J..

[21] Sevinc F., Cao S., Xuan X., Sevinc M., Ceylan O. (2015). Identification and expression of *Babesia ovis* secreted antigen 1 and evaluation of its diagnostic potential in an enzyme-linked immunosorbent assay. J. Clin. Microbiol..

[22] Ozubek S., Aktas M. (2017). Molecular and parasitological survey of ovine piroplasmosis, including the first report of *Theileria annulata* (Apicomplexa: Theileridae) in sheep and goats from Turkey. J. Med. Entomol..

[23] Oosthuizen M.C., Zweygarth E., Collins N.E., Troskie M., Penzhorn B.L. (2008). Identification of a novel *Babesia* sp. from a sable antelope (*Hippotragus niger* Harris, 1838). J. Clin. Microbiol..

[24] Aktas M., Altay K., Dumanli N. (2005). Development of a polymerase chain reaction method for diagnosis of *Babesia ovis* infection in sheep and goats. Vet. Parasitol..

[25] Almazán C., Bonnet S., Cote M., Slovák M., Park Y., Šimo L. (2018). A versatile model of hard tick infestation on laboratory rabbits. J. Vis. Exp..

[26] Frerichs W.M., Allen P.C., Holbrook A.A. (1973). Equine piroplasmosis (*Babesia Equi*): Therapeutic trials of imidocarb dihydrochloride in horses and donkeys. Vet. Rec..

[27] Ueti M.W., Mealey R.H., Kappmeyer L.S., White S.N., Kumpula-McWhirter N., Pelzel A.M., Grause J.F., Bunn T.O., Schwartz A., Traub-Dargatz J.L. (2012). Re-emergence of the apicomplexan *Theileria equi* in the United States: Elimination of persistent infection and transmission risk. Vet. Parasitol..

[28] Grause J.F., Ueti M.W., Nelson J.T., Knowles D.P., Kappmeyer L.S., Bunn T.O. (2013). Efficacy of imidocarb dipropionate in eliminating *Theileria equi* from experimentally infected horses. Vet. J..

[29] Butler C.M., Nijhof A.M., Van der Kolk J.H., De Haseth O.B., Taoufik A., Jongejan F., Houwers D.J. (2008). Repeated high dose imidocarb dipropionate treatment did not eliminate *Babesia caballi* from naturally infected horses as determined by PCR-Reverse Line Blot Hybridization. Vet. Parasitol..

[30] Cardillo N.M., Lacy P.A., Villarino N.F., Doggett J.S., Riscoe M.K., Bastos R.G., Laughery J.M., Ueti M.W., Suarez C.E. (2024). Comparative efficacy of buparvaquone and imidocarb in inhibiting the in vitro growth of *Babesia bovis*. Front. Pharmacol..

[31] Cardillo N.M., Villarino N.F., Lacy P.A., Riscoe M.K., Doggett J.S., Ueti M.W., Chung C.J., Suarez C.E. (2024). The combination of buparvaquone and ELQ316 exhibit a stronger effect than ELQ316 and imidocarb against *Babesia bovis* in vitro. Pharmaceutics.

[32] Cardillo N.M., Villarino N.F., Lacy P.A., Doggett J.S., Riscoe M.K., Suarez C.E., Ueti M.W., Chung C.J. (2025). Enhanced anti-*Babesia* efficacy of buparvaquone and imidocarb when combined with ELQ-316 in vitro culture of *Babesia bigemina*. Pharmaceuticals.

[33] Ozubek S., Ulucesme M.C., Aktas M. (2025). Transovarial transmission of *Babesia ovis* in *Rhipicephalus bursa*, confirmed by multi-generational experiments. Parasite.

[34] Kawahara M., Rikihisa Y., Lin Q., Isogai E., Tahara K., Itagaki A., Hiramitsu Y., Tajima T. (2006). Novel genetic variants of *Ana plasma phagocytophilum*, *Anaplasma bovis*, *Anaplasma centrale*, and a novel *Ehrlichia* sp. in wild deer and ticks on two major islands in Japan. Appl Environ Microbiol.

[35] Bekker C.P., De Vos S., Taoufik A., Sparagano O.A., Jongejan F. (2002). Simultaneous detection of *Anaplasma* and *Ehrlichia* species in ruminants and detection of *Ehrlichia ruminantium* in *Amblyomma variegatum* ticks by reverse line blot hybridization. Veterinary microbiology.

[36] Georges K., Loria G.R., Riili S., Greco A., Caracappa S., Jongejan F., Sparagano O. (2001). Detection of haemoparasites in cattle by reverse line blot hybridisation with a note on the distribution of ticks in Sicily. Vet. Parasitol..

