# Supplementary material for: Persistence and Transmission Dynamics of Babesia ovis After Imidocarb Dipropionate Treatment: Evaluation via Blood Transfusion and Tick Infestation
Source: Pathogens. 2025 Dec 20;15(1):7. doi: 10.3390/pathogens15010007 (PMC12844971; doi:10.3390/pathogens15010007)
Supplement: Supplementary file 1 [file pathogens-15-00007-s001.zip › pathogens-4049206-supplementary.pdf]

Supplementary Table S1: Primers and sequences used in this study for the detection of tick-borne pathogens

Tick-borne pathogens		Target gene	Primer	Primer sequence (5′–‘3)	Product size (bp)	Reference
Anaplasma / Ehrlichia	1st reaction	*16S rDNA*	Ec9	TACCTTGTTACGACTT	1462	[34]
			Ec12A	TGATCCTGGCTCAGAACGAACG		
	nPCR		16S8FE	GGAATTCAGAGTTGGATC(A/C)TGG(C/T)TCAG	492-498	[35]
			BGA1B	CGGGATCCCGAGTTTGCCGGGACTT(C/T)TTCT		
Babesia /Theileria	1st reaction	*18S rDNA*	Nbab1F	AAGCCATGCATGTCTAAGTATAAGCTTTT	1600	[23]
			Nbab1R	CTTCTCCTTCCTTTAAGTGATAAGGTTCAC		
	nPCR		RLBF2	GACACAGGGAGGTAGTGACAAG	390-430	[36]
			RLBR2	CTAAGAATTTCACCTCTGACAGT		
Babesia ovis	1st reaction	*18S rDNA*	Nbab1F	AAGCCATGCATGTCTAAGTATAAGCTTTT	1600	[23]
			Nbab1R	CTTCTCCTTCCTTTAAGTGATAAGGTTCAC		
	nPCR		BboF	TGGGCAGGACCTTGGTTCTTCT	549	[24]
			BboR	CCGCGTAGCGCCGGCTAAATA		
